# URG4 mediates cell proliferation and cell cycle in osteosarcoma via GSK3β/β-catenin/cyclin D1 signaling pathway

**DOI:** 10.1186/s13018-020-01681-y

**Published:** 2020-06-18

**Authors:** Yayun Liu, Yizhe Xi, Gang Chen, Xidong Wu, Maolin He

**Affiliations:** 1 Department of Spinal Surgery, The First Affiliated Hospital of Guangxi Medical University, No. 6 Shuangyong Road, Nanning, 530021 Guangxi China; 2 Department of Orthopaedics, Jiangxi Provincial People’s Hospital Affiliated to Nanchang University, No. 152 Aiguo Road, Nanchang, 330006 Jiangxi China; 3 Department of drug safety evaluation, Jiangxi Testing Center of Medical Device, No. 181 Nanjing East Road, Nanchang, 330000 Jiangxi China

**Keywords:** Upregulated gene 4, Osteosarcoma, GSK3β/β-catenin/cyclin D1, Cell proliferation, Cell cycle, Apoptosis

## Abstract

**Background:**

Osteosarcoma is one of the most common malignant bone tumors with the annual global incidence of approximately four per million. Upregulated gene 4 (URG4) expression in the osteosarcoma tissue is closely associated with recurrence, metastasis, and poor prognosis of osteosarcoma. However, the biological function and underlying mechanisms of URG4 in osteosarcoma have not been elucidated. This study aimed to explore the expression and underlying mechanism of URG4 in osteosarcoma.

**Methods:**

The expression level of URG4 in osteosarcoma and normal tissues was compared using immunohistochemistry (IHC). PCR and western blotting (WB) techniques are used to detect URG4 mRNA and protein levels. Wound healing and Transwell analysis to assess the effect of URG4 on osteosarcoma cell migration and invasion. Cell Counting Kit-8 assay and colony proliferation assay were performed to evaluate the effects of silencing URG4 on the inhibition of cell proliferation. The cell cycle distribution was detected by flow cytometry, and a xenograft mouse model was used to verify the function of URG4 in vivo.

**Results:**

URG4 was found to be highly expressed in osteosarcoma tissues and cells, and its high expression was correlated with advanced Enneking stage, large tumor size, and tumor metastasis in osteosarcoma patients. The proliferation in osteosarcoma cell lines and cell cycle in the S phase was suppressed when siRNA was used to downregulate URG4. URG4 promoted cell proliferation and tumorigenesis in vitro and in vivo. WB verified that URG4 promotes cell proliferation in osteosarcoma via pGSK3β/β-catenin/cyclinD1 signaling.

**Conclusion:**

URG4, which is high-expressed in osteosarcoma, promotes cell cycle progression via GSK3β/β-catenin/cyclin D1 signaling pathway and may be a novel biomarker and potential target for the treatment of osteosarcoma.

## Introduction

Osteosarcoma is one of the most common malignant bone tumors with the highest incidence in children and teenagers [1]. The annual global incidence is approximately four million. Since the early diagnosis of osteosarcoma is relatively rare, the combination of neoadjuvant chemotherapy and tumor resection is the main treatment for osteosarcoma, but it has a poor prognosis [2]. Despite improvements in treatment over the past few decades, it is still a tumor with a high mortality rate in metastatic or recurrent osteosarcoma patients with an overall survival rate of less than 20% [3, 4]. The poor prognosis of patients with osteosarcoma is due to unclear and controversial pathogenesis. Therefore, further study of factors related to osteosarcoma metastasis is warranted.

Tufan et al. originally identified the upregulated gene 4/upregulator of cell proliferation (URG4/URGCP) with subtractive hybridization and differential PCR technology [5]. URG4, a novel oncogene located on the short arm of chromosome 7p13, promotes cell proliferation via the MAPK, PI3K, Akt, and NF-kappa B pathways. Studies have shown that high levels of URG4 protein have been found in many solid tumors and are involved in tumorigenesis and tumor development, including hepatocellular carcinoma [6], gastric cancer [7], ovarian cancer [8], glioblastoma [9], glioma [10], breast carcinoma [11], non-small-cell lung cancer [12], and nasopharyngeal cancer [13]. Huang et al. suggested that URG4 expression in osteosarcoma tissue is closely associated with recurrence, metastasis, and poor prognosis of osteosarcoma [14]. Concretely, the survival time of patients with high URG4 expression is fairly short, indicating that URG4 may be a valuable prognostic indicator for osteosarcoma patients. It is worth noting that URG4 plays an important role in regulating the biological function of malignant tumors through a variety of signaling pathways (MAPK, PI3K, Akt, and NF-κB pathways). However, the biological function and underlying mechanisms of URG4 in osteosarcoma have not been elucidated.

Additionally, Glycogen Synthase Kinase-3β (GSK-3β) is involved in cell cycle progression and proliferation of multiple tumors, including osteosarcoma [15]. The involvement of the β-catenin/cyclin D 1 pathway in cell proliferation and differentiation consider as a crucial signaling pathway [16]. The presence of Wnt signal inhibits GSK-3β activity, and the degradation of β-catenin is interrupted [17]. The β-catenin accumulated in the cytoplasm is transferred to the nucleus, which activates Wnt target genes and various activating factors and promotes the expression of cyclin D1. Therefore, this signaling pathway plays an important role in tumor progression and metastasis. Whether or not URG4 is involved in the biological behavior of osteosarcoma cells through the GSK-3β/β-catenin pathway is still unknown.

In the present study, URG4 is highly expressed in osteosarcoma cells and is closely associated with tumor development and clinical typing. Moreover, URG4 regulates cell cycle and proliferation in osteosarcoma through the GSK3β/β-catenin/cyclin D1 pathway. This URG4 may become a clinical marker for the diagnosis of osteosarcoma, and the results of the current study may provide the theoretical basis for diagnosis and treatment.

## Materials and methods

### Tissue sample collection

These experiments of animals and humans were authorized by the ethics committee of Jiangxi Provincial People’s Hospital. From April 2012 to June 2017, forty primary osteosarcoma tissues with paired corresponding normal tissues were included in the present study with the patients’ consent in Jiangxi Provincial people’s Hospital. For immunohistochemical analysis, all the tissues were fixed with 10% formalin, embedded in paraffin, and serially sectioned at a thickness of 4 microns. The detailed clinicopathological information is shown in Table 1.

**Table 1:** Correlation analysis between URG4 protein expression and clinicopathological variables

Variable	*N*	URG4 expression		*χ*^2^	*P*
		Low	High		
Gender					
Male	29	9	20	0.729	0.393
Female	11	5	6		
Age					
<18 years	31	10	21	0.455	0.499
≥18 years	9	4	5		
Location					
Femur	21	6	15	0.806	0.668
Tibia	12	5	7		
Other sites	7	3	4		
Tumor necrosis rate (%)					
<90	35	12	23	0.063	0.802
≥90	5	2	3		
Tumour size (cm)					
<5	26	12	14	4.062	0.043
≥ 5	14	2	12		
Histologic type					
Osteoblastic	31	9	22	2.157	0.142
Chondroblastic	9	5	4		
Tumour metastasis					
Presence	13	1	12	6.313	0.012
Absence	27	13	14		
Enneking stages					
I	9	7	2	9.455	0.009
II	25	6	19		
III	6	1	5		

### Immunohistochemistry (IHC)

The histologic sections were independently evaluated by two pathologists who were blinded to the clinical data. Paraffin-embedded tissue sections were used to detect URG4 expression. Sections from each sample were first subjected to hematoxylin and eosin (HE) staining to determine the pathological diagnosis, and the subsequent slide was conventionally dewaxed and rehydrated for further immunohistochemical staining. Slices were incubated with a URG4 primary antibody (1:100, Cat NO: 11998–1-AP, Proteintech, Wuhan, China) overnight at 4 °C, followed by further incubation with a secondary antibody (1:200, Cat NO: GB23303, Servicebio, Wuhan, China) at room temperature for 40 min. The sections were then immersed in diaminobenzidine, counterstaining with hematoxylin. The Olympus microscope (Tokyo microscope, Japan) was used to observe and photograph the staining signals, and the staining indexes were measured and calculated using Olympus FV10-ASW software. A negative control comprising treatment with phosphate-buffered saline (PBS) instead of the primary antibody was performed with all the tissue samples. The staining intensity of URG4 was ranked at four levels: 0 (negative), 1 (weak), 2 (moderate), or 3 (strong), and the percentage of positively stained cells was scored as 0 (≤ 5%), 1 (6–25%), 2 (26–50%), 3 (51–75%), 4 (> 75%). The product of the staining intensity and percentage constituted the final staining score (0–12), and the rankings were transformed into a sum index as follows: −, score 0; +, score 1–4; ++, score 5–8; and +++, score 9–12.

### Cell culture and lentivirus infection

Five osteosarcoma cell lines (MNNG/HOS, 143B, U2OS, Saos-2, and MG63) and a human osteoblast cell line (hFOB 1.19) were purchased from Shanghai Genechem Company Ltd. U2OS, 143B, and MG63 cells were cultured in DMEM (Gibco, USA), whereas MNNG/HOS and Saos-2 cells were cultured in RPMI 1640 medium. hFOB 1.19 cells were cultured in 1:1 DMEM/Ham’s F12 medium supplemented with 2.5 mM l-glutamine and 0.3 mg/ml G418 (Sigma-Aldrich, St Louis, MO, USA). All medium contained 10% fetal bovine serum (FBS) (Biowest, South America), 100 U/mL penicillin, and 0.1 mg/mL streptomycin (Leagene Biotechnology, Beijing, China). All cells were incubated at 37 °C containing 5% CO_2_.

Lentivirus expressed siRNA targeting URG4 (siURG4) or a nonspecific control siRNA (si-NC) was synthesized by Shanghai Genechem Company Ltd. For transfection, two target cell lines, HOS and MG63 cells, were seeded in twelve-well plates and then transfected with lentivirus containing either one of two different siURG4 sequences or si-NC. The transduced cells were screened with puromycin, and the objective gene silencing efficiency was assessed by qRT-PCR and western blot (WB) analysis.

### RNA extraction and qRT-PCR analysis

Total RNA was extracted with TRIzol reagent (Invitrogen, Carlsbad, CA, USA) according to the manufacturer’s instructions. URG4 mRNA expression levels were detected by quantitative real-time PCR using PrimeScript® RT (Takara Biotech, Dalian, China) with GAPDH as an internal control. The primer sequences were as follows: URG4 (forward 5′-TGCTGCCGACATTTATTCCTT-3′, reverse 5′-GGCCATTTTCAACGCTATTTCT-3′); and the control (forward 5′-TCGACAGTCAGCCGCATCT-3′, reverse: 5′-CTTGACGGTGCCATGGAATT-3′). Relative quantification was conducted using the 2-ΔΔCt method.

### Protein extraction and WB analysis

Cells in the exponential growth period were collected and lysed on ice in lysis buffer (cw0048S, Kangweishiji Biotechnology, Beijing, China) containing protease and phosphatase inhibitors. The protein concentration was tested using the bicinchoninic acid (BCA) method (cw0014S, Kangweishiji Biotechnology, Beijing, China). Equal amounts of protein from each sample were separated by SDS-PAGE on the separating gel and transferred onto PVDF membranes (Basel, Switzerland, Roche). Then, the membranes were blocked by using 5% skimmed milk and incubated overnight with primary antibodies [URG4 (1:1,000, Cat NO: 11998–1-AP, Proteintech, Wuhan, China); β-catenin (1:7,000, Cat NO: ab32572, Abcam Co. Ltd.); GSK3β (1:7,000, Cat NO: ab32391, Abcam Co. Ltd.); P-GSK3β^ser9^ (1:16,000, Cat NO: ab75814, Abcam Co. Ltd.); cyclin D1 (1:10,000, Cat NO: ab134175, Abcam Co. Ltd.); Histone H3 (nuclear loading control, 1:2,000, Cat NO: ab176842, Abcam Co. Ltd.); and β-actin (mouse monoclonal, 1:2,000, Cat NO: T0022, Affinity Biosciences)] at 4 °C. The next day, the membranes were incubated with secondary antibody [anti-rabbit IgG (1:5,000, cw0103S, Kangweishiji Biotechnology Co. Ltd., Beijing, China)] for 1 h. The signal was observed via enhanced chemiluminescence by mixing EclA and EclB solution, after which the membranes were finally developed and fixed. The relative amount of protein was calculated by measuring the density of each protein band using ImageJ software (Barcelona, Spain). β-Actin (1:2,000, Cat NO: T0022, Affifinity) and histone H3 (1:2,000, Cat NO: ab176842, Abcam Co. Ltd.) were used as internal parameters.

### Nuclear protein extraction and immunofluorescence staining

Nuclear β-catenin protein was extracted using a nuclear protein extraction kit according to the manufacturer’s instructions. Cell suspensions of each group of MG63 and HOS cells were seeded on coverslips in a six-well plate and routinely incubated for approximately 2 h. Next, the cells were incubated with 2 ml of culture medium for 6 h. After having been washed 3 times with PBS, the cells were fixed with 4% paraformaldehyde for 20 min, followed by another washing 3 times with PBS. The coverslip was immersed in a BSA solution blocking for 30 min. Then, they were treated dropwise with prepared primary antibody β-catenin (1:200, Cat NO: GB13015, Servicebio, Wuhan, China) and incubated overnight at 4 °C. After washing 3 times with PBS for 5 min per wash, treated dropwise with the appropriate fluorescent secondary antibody: Cy3 goat anti-rabbit (1:300, Cat NO: GB21303, Servicebio, Wuhan, China) and incubated under dark environment for 50 min, followed by further incubation with 4′,6-diamidino-2-phenylindole (DAPI) for 10 min at room temperature. Sections were viewed through a Nikon upright fluorescence microscope, and images were acquired.

### Cell proliferation and colony formation assays

Cell Counting Kit-8 (CCK8) and colony formation assays were used to detect cell proliferation. The transduced stable cell lines (MG63 and HOS) were digested with trypsinase and seeded into 96-well plates (approximately 2000 cells per well) with seven replicates in each group. Cell viability at various time points (24, 48, 72, and 96 h after planting) was analyzed by CCK8 (CCK8: Dojindo Laboratories, Japan). In the colony formation experiment, MG63 and HOS cells during the exponential growth period were seeded in a six-well plate (1000 cells per well) and routinely cultured with three replicates. When cultures were observed, they were washed twice with PBS and then fixed with 4% paraformaldehyde for 15 min. Cultures were stained with GIMSA for 20 min and washed again. Colonies whose cell counts exceeded 30 were recorded by an inverted microscope.

### Cell migration and invasion assays

The migration ability of cells was measured by a wound healing test. Approximately 5 × 10^5^ cells were added into each well with MG63 or HOS cells stably expressing control siRNA, siRNA1-URG4, or siRNA2-URG4 and cultured under permissive conditions until 90% confluence. The confluent cell monolayer was lightly and quickly scratched with a pipette tip perpendicular to produce a straight line. The detached cells were removed with PBS washing, treated with serum-free medium, and cultured in an incubator at 37 °C containing 5% CO_2_. Representative photographs were taken under a microscope at 100-fold magnification after scratching 0, 12, and 24 h. Mobility was assessed as observed width of the wound/primary width.

Transwell inserts (6.5 mm, Corning, cat. no. 3422, Corning, NY, USA) were placed in a 24-well plate to assess invasion. Matrigel (BD Co., cat. no. 356234, Bedford, MA, USA) was diluted 1:8 with serum-free medium, 80 μl of which was added into the upper chamber of each transwell and incubated at 37 °C for at least 2 h. Approximately 200 μl MG63 or HOS cells (5 × 10^5^ cells/ml) were added into 200 μl medium containing 5% FBS in each upper chamber, and 500 μl of complete medium containing 20% FBS was added to the lower chambers. After 48 h incubation at 37 °C containing 5% CO_2_, the invading cells were fixed with anhydrous formaldehyde and stained with crystal violet. Five randomly selected fields of view were observed and photographed.

### Apoptosis assay

Apoptosis was measured by an Annexin V/FITC Apoptosis Assay Kit (Bestbio, Shanghai, China) using a FACSCalibur flow cytometer (BD Biosciences, San Jose, CA, USA). MG63 or HOS cells expressing negative control or URG4 siRNA were digested in EDTA-free 0.25% trypsin to obtain a single-cell suspension. The cells were collected by centrifugation at 1500 rpm for 5 min, labeled with 100 μl 1× Binding buffer containing 5 μl annexin V/FITC and 5 μl propidium iodide (PI), and then samples were incubated at room temperature for 15 min in the dark. Data were collected by flow cytometry within 1 h. Each group was repeated at least 3 times.

### Cell cycle analysis

MG63 and HOS cells were collected, resuspended in PBS at 4 °C and centrifuged at 1500 rpm for 5 min. After the supernatant was discarded, 75% ethanol that was pre-cooled at − 20 °C was slowly added to the pellet, which was then incubated overnight at 4 °C. Cells were washed with PBS and resuspended, adding 1 mg/ml RNase A, and samples were incubated at 37 °C for 40 min. After 100 μl of 100 g/ml PI staining solution (Kaiji, China) addition, another incubation for 20 min in the dark was performed. The cell cycle distribution was tested by flow cytometry and analyzed using Modfit software.

### Xenograft tumor formation in a nude mouse model

Six-week-old male BALB/c nude mice were purchased from the Hunan Slack Jingda Experimental Animal Co., Ltd. (Changsha, China). Our all animal experiment accepted the supervision and inspection of the Ethics committee of Jiangxi Provincial People’s Hospital. HOS and MG63 cells (1 × 10^7^ cells/ml in PBS) transfected with siURG4-1 or NC siRNA were subcutaneously injected into nude mice (0.1 ml). Tumor size was assessed at 4, 7, 13, 19, 25, and 31 days after inoculation with the following formula: *V* (volume) = (length × width^2^)/2. At 31 days post-inoculation, all mice were euthanized, and tumors were collected and weighed.

### Statistical analysis

The results of this study were analyzed by SPSS version 20.0 (SPSS, Inc., Chicago, IL, USA), and values were expressed as the mean ± standard deviation (SD) at least three different experiments. A double tail Student’s *t* test was used to compare the differences between groups. The correlation between the immunohistochemical results and clinicopathological parameters was examined by the chi-square test. A value of *P* < 0.05 was considered statistically significant.

## Results

### Increased expression of URG4 in human osteosarcoma cell lines and tissues

**Fig. 1:**
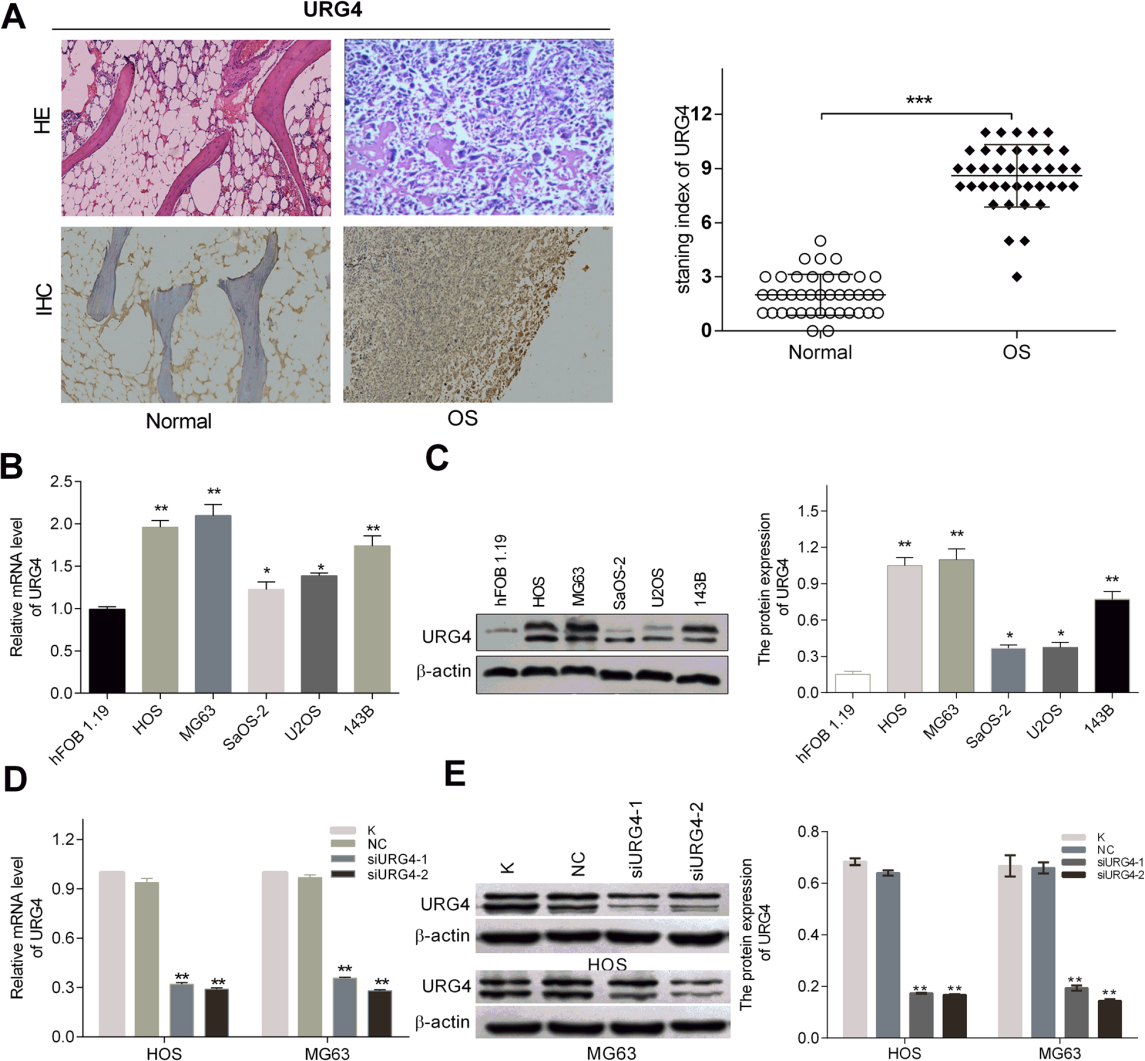
Increased expression of URG4 in osteosarcoma tissues and cell lines. **a** URG4 expression was significantly increased in osteosarcoma tissues than corresponding normal tissues by HE and IHC, respectively (× 200 magnification). **b** PCR determined URG4 mRNA expression in osteosarcoma cell lines (HOS, MG63, Saos-2, U2OS, and 143B), and hFOB 1.19 was used as control. **c** Western blot assay determined URG4 protein expression in osteosarcoma cell lines (HOS, MG63, Saos-2, U2OS, and 143B), and hFOB 1.19 was used as control. **d** The mRNA expression level of the URG4 in HOS and MG63 cell lines following transfection as determined by RT-qPCR. **e** The protein expression level of URG4 in HOS and MG63 cell lines following transfection as determined by western blot assay. HE: hematoxylin and eosin; IHC: Immunohistochemistry; URG4: upregulated gene 4; Normal: normal tissues; OS: osteosarcoma tissues; K: blank group; NC: negative control. *p < 0.05, **p < 0.01 vs the NC

To investigate the role of URG4 in osteosarcoma, the IHC method was performed to compare the expression level of URG4 in osteosarcoma and normal tissues. URG4 expression in osteosarcoma tissue was significantly higher than that in normal tissue (Fig. 1a). The correlation between URG4 expression and clinicopathological characteristics of 40 patients with osteosarcoma was shown in Table 1. Our results reveal that URG4 expression was closely related to tumor size (*χ*^2^ = 4.062, *p* = 0.043), tumor metastasis (*χ*^2^ = 6.313, *p* = 0.012), and Enneking stage (*χ*^2^ = 9.455, *p* = 0.009). Meanwhile, we used PCR and WB techniques to detect URG4 mRNA and protein levels, respectively. The mRNA levels of URG4 were increased significantly in the human osteosarcoma cell lines HOS, MG63, Saos-2, U2OS, and 143B compared to hFOB 1.19 cells (Fig. 1b). The levels of protein were also increased significantly in the human osteosarcoma cell lines compared to hFOB 1.19 cells (Fig. 1c). These results showed that URG4 is upregulated in osteosarcoma tissues and cell lines, suggesting that URG4 may play an important role in the occurrence and development of osteosarcoma.

### URG4 downregulation inhibited the migration and invasion of osteosarcoma cells

To study the functional significance of URG4 in osteosarcoma, HOS and MG63 cells were selected owing to their relatively higher expression of URG4 and treated with siRNAs targeting URG4 to downregulate URG4 expression in osteosarcoma cells. RT-qPCR and WB analysis were employed to compare the expression levels of URG4 in HOS and MG63 cell lines followed by transfection and the blank group and negative control group. In both cell lines, the mRNA and protein expression of URG4 decreased significantly after transfection with siURG4 (Fig. 1d, e), verifying its downregulation effect.

**Fig. 2:**
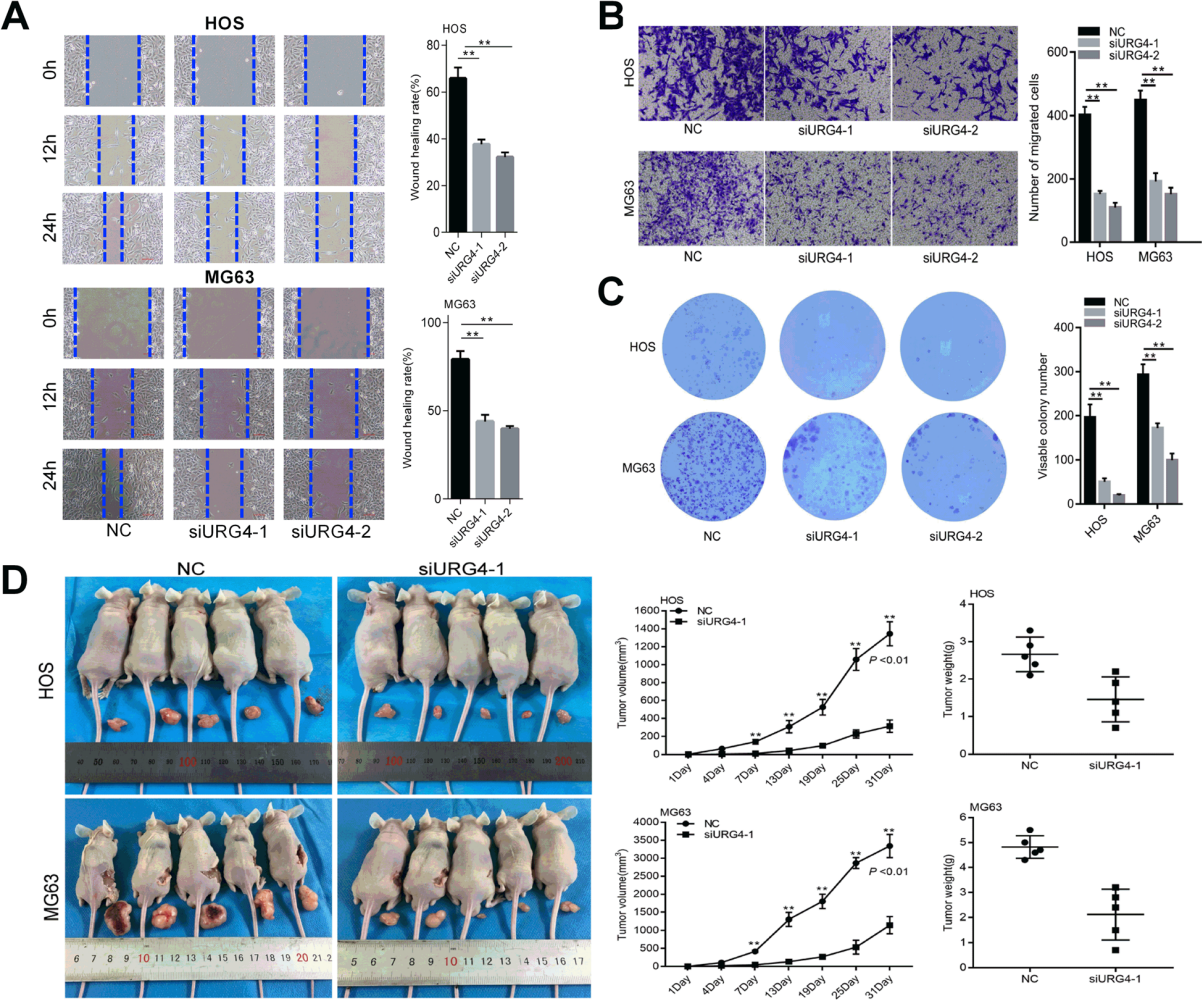
URG4 affects vitro migration, invasion, colony formation, and vivo tumorigenesis. **a** HOS and MG63 cells stably transfected with siURG4 or siCtrl, and migration ability was evaluated by wound-healing assay. **b** Transwell assay was performed to analyze invasive ability. **c** Colony-forming abilities were researched by plate clones assay. **d** Images, volume, and weight of transplanted tumors in nude mice 31 days after subcutaneous inoculation with osteosarcoma cells stably expressing siURG4-1 or si-NC. URG4: upregulated gene 4; K: blank group; NC: negative control. *p < 0.05, **p < 0.01 vs the NC

The wound-healing assay revealed that the number of cells migrating through the wound area in the siRNA1-URG4 and siRNA2-URG4 groups was decreased significantly compared with that in the negative control group (NC) group 24 h after scratching the HOS and MG63 cell monolayers (Fig. 2a). As shown in Fig. 2b, MG63 and HOS cells with siRNA targeting URG4 have an ameliorated invasion ability vs cells with the NC group by Transwell assays. These results suggested that URG4 gene downregulation suppresses the migration and invasion of osteosarcoma cells.

### URG4 downregulation inhibited the proliferation of osteosarcoma cells

**Fig. 3:**
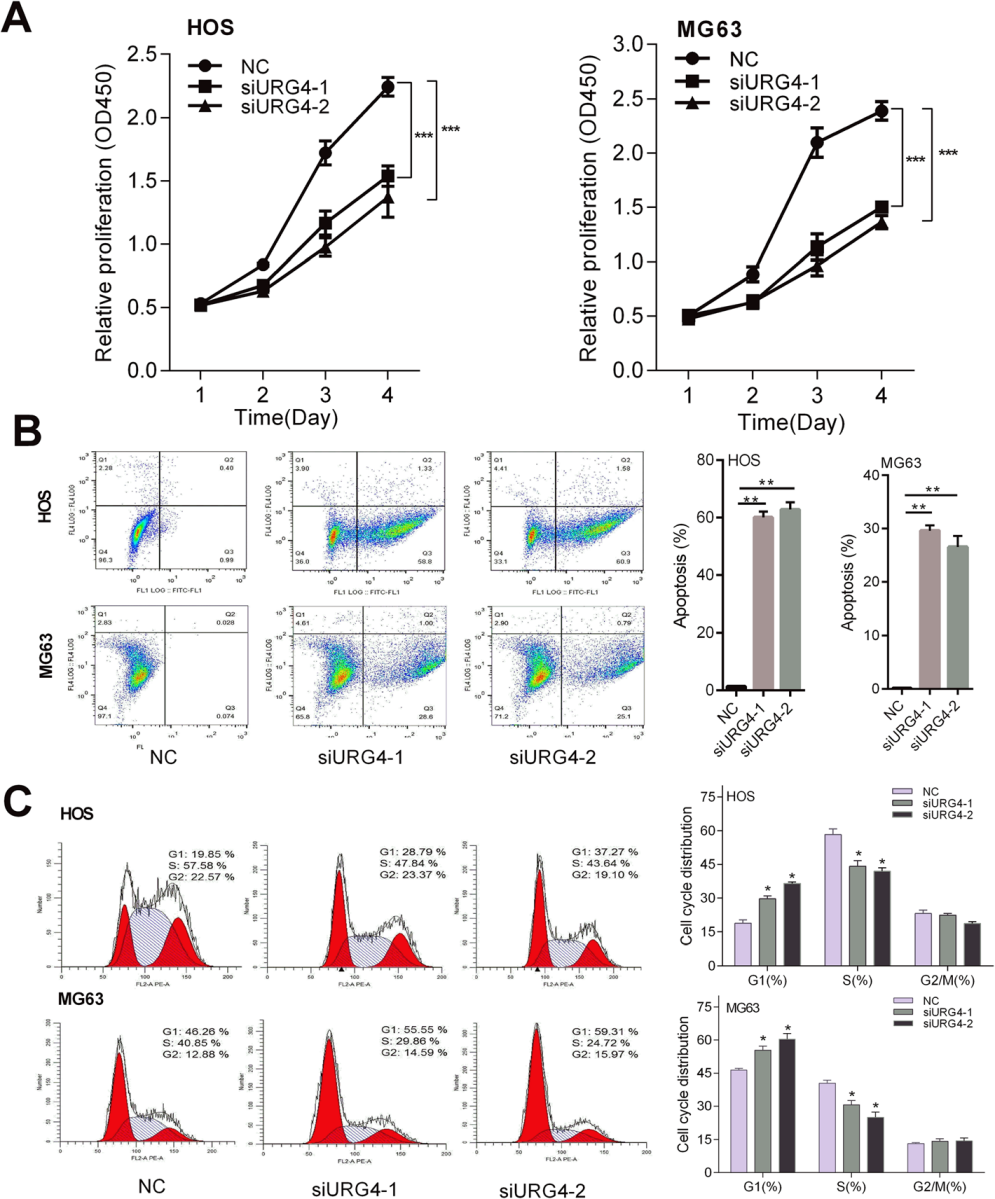
URG4 mediates proliferation, apoptosis, and cell cycle in vitro. **a** The proliferative capacity of cells following transfection was measured by CCK8 assay. **b** The apoptosis rate of HOS and MG63 cells was detected by flow cytometry. **c** The DNA content of PI-stained HOS and MG63 cells at the G0/G1, S, and G2/M phases was assessed by flow cytometry. CCK8: Cell Counting Kit-8; URG4: upregulated gene 4; NC: negative control. *p < 0.05, **p < 0.01, ***p < 0.001 vs the NC

To assess the effects of silencing URG4 on the inhibition ability of cell proliferation, Cell Counting Kit-8 assay and the colony proliferation assay were performed in osteosarcoma cells. As shown in Fig. 2c, colony formation abilities of HOS and MG63 cells were strongly inhibited after URG4 was silenced compared with the NC group. Moreover, suppression of URG4 exhibited marked inhibition in osteosarcoma cell proliferation compared to the NC group from day 3 to day 4 (Fig. 3a).

### URG4 downregulation promoted osteosarcoma cell apoptosis

To reveal whether the downregulation of the URG4 could affect rates of osteosarcoma cell apoptosis, flow cytometry assay was carried out in si-URG4 cells. The flow cytometry data illustrated that the apoptosis rate of two osteosarcoma cell lines (MG63 and HOS) after transfection with siRNA1-URG4 and siRNA2-URG4 was relatively higher than that of the NC group (Fig. 3b). Our results revealed that URG4 downregulation promoted cell apoptosis in osteosarcoma cells.

### URG4 downregulation influenced the progression of osteosarcoma cell cycle

To further identify the role of URG4 downregulation in the progression of the osteosarcoma cell cycle, the cell cycle distribution was detected by flow cytometry. Cell cycle analysis showed that HOS and MG63 cells processed with siRNA1-URG4 or siRNA2-URG4 had a significant increase of cells in the G1 phase and a decrease of cells in S phase (Fig. 3c). These findings demonstrated that silenced URG4 increased the percentage of cells in the G1 phase and reduced that of cells in the S phase, representing that downregulating URG4 might be responsible for influences in the osteosarcoma cell cycle.

### URG4 downregulation inhibited the tumorigenicity of osteosarcoma in vivo

A xenograft mouse model of osteosarcoma was used to verify the carcinogenic effect of URG4 in vivo. HOS and MG63 cells transfected with NC or siRNA1-URG4 lentivirus were injected either under the right axillary region or in the right hip of nude mice, and their overall health status and tumor development after cell transplantation were observed. The results showed that the average size and weight of tumors in the NC group were clearly higher than those in the siRNA1-URG4 group (Fig. 2d), which indicates that silencing URG4 can inhibit the tumorigenic effects of osteosarcoma cells in vivo.

### URG4 promotes osteosarcoma cell proliferation through the GSK3β/β-catenin/cyclin D1 signaling pathway

**Fig. 4:**
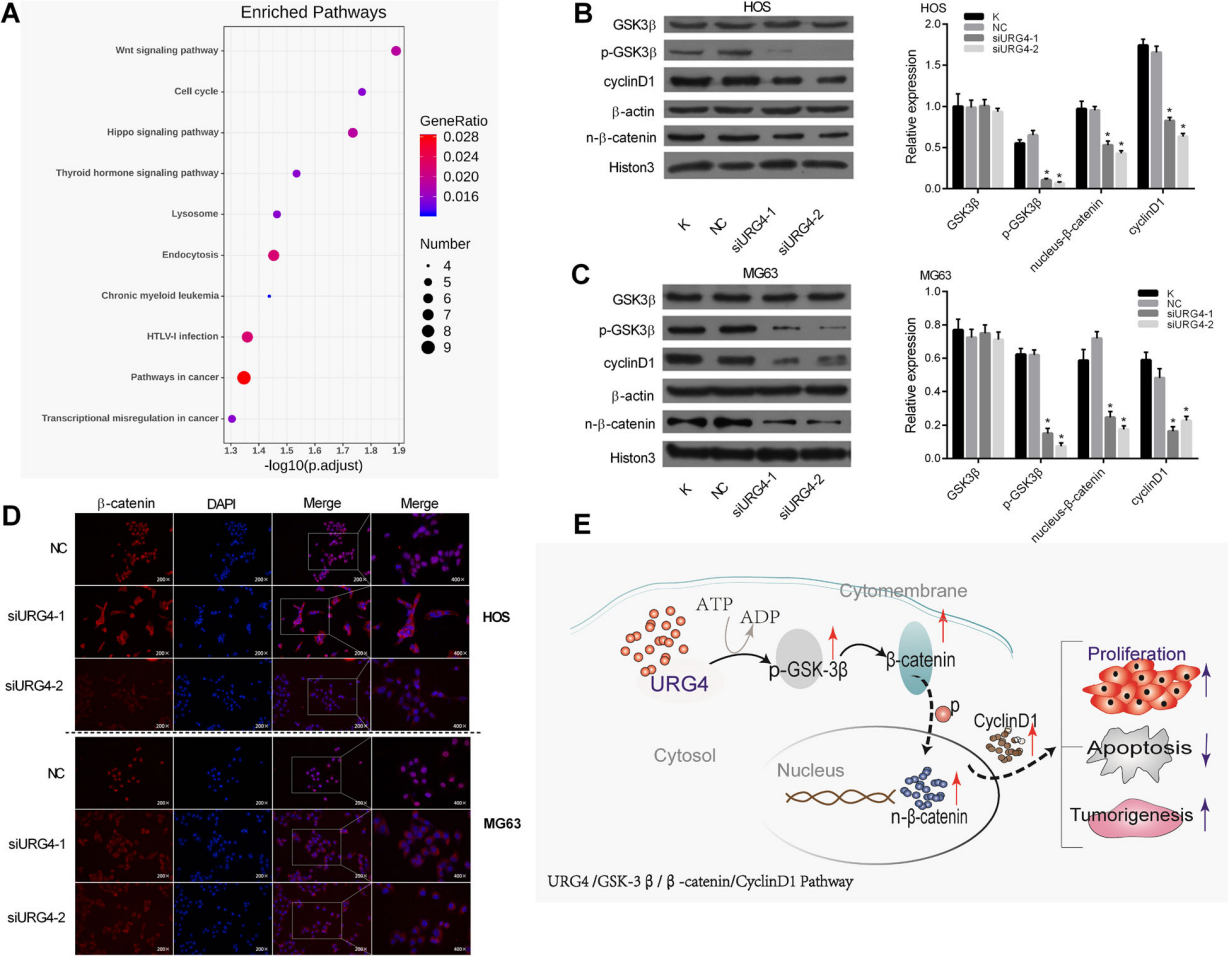
URG4 regulates osteosarcoma cell growth by activating the GSK3β/β-catenin/cyclin D1 signaling pathway. **a** URG4-related genes enriched in the top 10 significant signaling pathway based on KEGG. **b** The protein level of GSK3β, p-GSK3β, nuclear β-catenin, and cyclin D1 in the HOS cell transfected with siURG4 was revealed by the western blot assay. **c** The protein level of GSK3β, p-GSK3β, nuclear β-catenin, and cyclin D1 in the MG63 cell transfected with siURG4 was revealed by the western blot assay. **d** The positive expression of the nuclear β-catenin protein in osteosarcoma cells was measured by the immunohistochemistry assay. **e** A model depicted URG4 promotes osteosarcoma progression via the GSK3β/β-catenin/cyclin D1 pathway. URG4: upregulated gene 4; NC: negative control. *p < 0.05 vs the NC

To investigate key signaling molecules influencing URG4-mediated changes in cell cycle progression and proliferation of human osteosarcoma, we performed the Kyoto Encyclopedia of Genes and Genomes (KEGG) pathway enrichment analysis and it was found that there were 10 significant signaling pathways related to URG4 (Table 2 and Fig. 4a). We found that the URG4-related genes enriched in the Wnt signaling pathway were the most significant (*p* = 0.012891). Thus, the protein levels of total GSK 3β, phosphorylated GSK 3β^ser9^, nuclear β-catenin, and cyclin D1, which are the key signaling molecules in the Wnt signaling pathway, were measured in HOS and MG63 osteosarcoma cells transfected with siRNA1-URG4, siRNA2-URG4, NC, and blank by WB. As shown in Fig. 4b, c, compared with the blank and NC groups, the expression levels of phosphorylated GSK 3β^ser9^, cyclin D1, and nuclear β-catenin in siRNA-URG4 groups were decreased, while that of total protein GSK 3β was unchanged. Additionally, changes of nuclear β-catenin in osteosarcoma cells were observed using nuclear staining. IHC analysis showed that the downregulation of URG4 expression significantly reduced nuclear β-catenin expression in HOS and MG63 cells (Fig. 4d). A previous study showed that inhibition of GSK 3β in the pathway contributes to the accumulation of nuclear β-catenin, which affected cell proliferation [18]. Altogether, GSK 3β/β-catenin/cyclin D1 signaling is involved in URG4-mediated osteosarcoma cell proliferation (Fig. 4e).

**Table 2:** KEGG enrichment analysis of URG4-related gene

Category	Term	Count	*P* value
KEGG_PATHWAY	hsa04310:Wnt signaling pathway	6	0.012891
KEGG_PATHWAY	hsa04110:Cell cycle	5	0.017052
KEGG_PATHWAY	hsa04390:Hippo signaling pathway	6	0.018397
KEGG_PATHWAY	hsa04919:Thyroid hormone signaling pathway	5	0.029209
KEGG_PATHWAY	hsa04142:Lysosome	5	0.034315
KEGG_PATHWAY	hsa04144:Endocytosis	7	0.035249
KEGG_PATHWAY	hsa05220:Chronic myeloid leukemia	4	0.036543
KEGG_PATHWAY	hsa05166:HTLV-I infection	7	0.043769
KEGG_PATHWAY	hsa05200:Pathways in cancer	9	0.04501
KEGG_PATHWAY	hsa05202:Transcriptional misregulation in cancer	5	0.049684

## Discussion

Osteosarcoma originates from mesenchymal malignant tumors and is the most commonly observed initial malignancy in orthopedics [19]. Highly aggressive and easy to early metastatic features in osteosarcoma are the main reasons for poor treatment and death of osteosarcoma patients [20, 21]. Recently, researchers have shown that the occurrence and progress of osteosarcoma have a close relationship with the aberrant activation of some proto-oncogenes, the abnormal inactivation of tumor suppressor genes, and the abnormal signaling of regulatory factors [22]. URG4, a new gene on chromosome 7, may play an important role in the development of osteosarcoma [14]. However, as etiology and pathogenesis of osteosarcoma are still unclear, current treatment strategies still face challenges. Therefore, identifying the role of URG4 in osteosarcoma is crucial for developing new effective treatment strategies. In this study, our results showed that URG4 was upregulated in osteosarcoma tissues and cell lines and associated with tumor stage.

As an oncogene of osteosarcoma, URG4 was highly expressed in primary osteosarcoma tissues, and its expression level was significantly relevant to the tumor size, tumor stage, and metastasis rate. Huang et al. confirmed that URG4 expression in normal tissue was relatively lower than that in osteosarcoma tissue and the survival rate of patients with high URG4 expression was significantly lower than that of patients with low URG4 expression [14], which is consistent with our study. These data indicate that URG4 plays an important role in the occurrence and development of osteosarcoma. URG4 is one of the downstream genes of hepatitis B virus X protein (HBx) that promotes transcription as a trans-activator and is named for its upregulation of cell proliferation. URG4 expression is mainly regulated by DNA methylation, transcription factors, and drugs at the transcriptional level. URG4 is associated with the occurrence, development, treatment, and prognosis of various cancers such as nasopharyngeal carcinoma [23], cervical cancer [24], and gastric cancer [7]. There is evidence that URG4 plays a carcinogenic role in the progress of malignant tumors by promoting the growth, proliferation, invasion, and metastasis of tumor cells and inhibiting apoptosis [25–27].

One of the central findings of the current study was that increased cell proliferation activity can enhance the progression of tumors [28]. Moreover, our studies demonstrated that silencing of URG4 contributes to the significant inhibition of proliferation in osteosarcoma cells and suppression of xenograft tumors in nude mice. It is known that apoptosis and the cell cycle are closely related to the proliferation of tumors [28–30]. Our results confirmed that URG4 downregulation in osteosarcoma cells can accelerate apoptosis, increase the number of cells in G1 phase, thereby reducing the number of cells in S phase. The regulation of G1/S transition is the most important checkpoint in the cell cycle. There are mainly three types of cyclins in G1 phase, namely, cyclin C, D, and E, and cyclin D1 plays a major role at this stage. URG4 promotes cell growth by upregulating cyclin D1. Satiroglu-Tufan et al. demonstrated that cyclin D1 plays a vital role in the mechanism of URG4-/URGCP-mediated liver cell growth [31]. Xie et al. further reported that overexpression of URG4 protein in hepatoma cells promotes cells rapidly entering the S phase, thus stimulating cell growth and proliferation through the increase of protein cyclin D1 [32]. Dodurga et al. also observed similar results in prostate cancer, in which the URG4 overexpression of androgen-dependent prostate cancer LNCAP cell lines increases cyclin D1 expression [33]. Recent studies have found that inhibition of URG4 may downregulate the expression of cyclin D1, resulting in cell cycle arrest and suppression of cell proliferation [34, 35]. These findings are consistent with the results of our URG4 gene downregulation experiments—in vitro proliferation of osteosarcoma cells (HOS, MG63) and in vivo tumorigenesis in nude mice. It was found that proliferating cell nuclear antigen (PCNA), a proliferation-related marker in neoplasms, had its maximum expression peak in the S phase and G2 phase of the cell cycle [36, 37]. In the previous study, we analyze that the high expression level of URG4 can stimulate cell growth and promote cells to enter the S phase through the cell cycle-related protein cyclin D1. Our results suggest that URG4 is an important oncogene, and its expression level is closely linked to the occurrence and development of osteosarcoma.

GSK-3β, a serine/threonine protein kinase, is now considered to be a regulator of a variety of cellular functions and can phosphorylate and regulate a variety of signaling proteins and transcription factors [38]. A large number of studies have investigated the potential pathways by which UGR4 regulates tumor cell proliferation and cell cycle progression. Several studies have revealed that UGR4 could control the proliferation and cell cycle progression of tumor cells by regulating cyclin D1 [10, 23]. An increase in cell transcription is usually accompanied by an increase in protein translation and a decrease in hydrolysis; thereby, the stability of cyclin D1 is a key factor affecting cell proliferation [39]. GSK-3β can target cyclin D1 and β-catenin by virtue of its kinase activity to achieve ubiquitin-dependent proteasome degradation [40]. Our study found that the level of phosphorylated GSK-3β^ser9^ decreased while total GSK-3β expression was not significantly changed by URG4 downregulation. Consistently, we found that nuclear β-catenin expression in HOS and MG63 cell decreased with a decrease in URG4 expression. Xie et al. found that the level of phosphorylated GSK-3β was increased by overexpression of URG4 in hepatoma cells [32]. Since the transcription of cyclin D1 protein can be upregulated by β-catenin signaling, β-catenin is crucial for cell proliferation [41, 42], and our current study has demonstrated that URG4 enhanced the stability of cyclin D1 by inducing the phosphorylation of GSK-3β resulting in the accumulation of β-catenin and its nuclear translocation. The accumulation of nuclear β-catenin promotes cyclin D1 expression, which affects cell proliferation and the cell cycle. So we conclude that URG4 can inactivate GSK-3β, thereby activating the β-catenin/cyclin D1 signaling pathway and promoting osteosarcoma cell proliferation. The exact mechanism by which URG4 is involved in GSK 3β/β-catenin/cyclin D1 pathway requires further investigation. Additionally, β-catenin can also be activated in osteosarcoma, which produces a considerable effect on tumor proliferation and epithelial-mesenchymal transition (EMT) [43]. In this study, we also found that the migration and invasion of osteosarcoma cells were significantly inhibited by URG4 downregulation through wound healing and Transwell experiments, respectively. It is speculated that URG4 may activate EMT by acting on β-catenin, thereby promoting the migration and invasion of osteosarcoma cells, but its mechanism remains to be further elucidated.

## Conclusion

In this study, we demonstrated that URG4 is highly expressed in osteosarcoma cells and clinical tissues, which is closely linked with tumor development and clinical typing. In addition, URG4 is an important regulator of proliferation, apoptosis, and invasion in osteosarcoma cells. Specifically, we found that URG4 regulates the cell cycle through the GSK 3β/β-catenin/cyclin D1 pathway, thereby affecting the proliferation of osteosarcoma cells. Data suggests that URG4 may be not only a predictor of osteosarcoma but also a potential target for osteosarcoma treatment.

## Data Availability

All data generated or analyzed during this study are included in this published article.
